# Intra-Individual Variability from a Lifespan Perspective: A Comparison of Latency and Accuracy Measures

**DOI:** 10.3390/jintelligence6010016

**Published:** 2018-03-14

**Authors:** Delphine Fagot, Nathalie Mella, Erika Borella, Paolo Ghisletta, Thierry Lecerf, Anik De Ribaupierre

**Affiliations:** 1 Center for the Interdisciplinary Study of Gerontology and Vulnerability (CIGEV), University of Geneva, 1211 Geneva, Switzerland; Anik.deRibaupierre@unige.ch; 2 Swiss National Centre of Competence in Research LIVES–Overcoming Vulnerability, Life Course Perspectives, University of Geneva, 1211 Geneva, Switzerland; Paolo.Ghisletta@unige.ch; 3 Cognitive Aging Lab, University of Geneva, 1211 Geneva, Switzerland; Nathalie.Mella-Barraco@unige.ch; 4 Department of General Psychology, University of Padova, 35122 Padova, Italy; erika.borella@unipd.it; 5 Faculty of Psychology and Educational Sciences, University of Geneva, 1211 Geneva, Switzerland; Thierry.Lecerf@unige.ch; 6 Swiss Distance Learning University, 3900 Brig, Switzerland; 7 Faculty of Social and Political Sciences, University of Lausanne, 1015 Lausanne, Switzerland

**Keywords:** intra-individual variability, working memory, life-span, reaction time

## Abstract

Within-task variability across trials (intra-individual variability (IIV)) has been mainly studied using latency measures but rarely with accuracy measures. The aim of the Geneva Variability Study was to examine IIV in both latency and accuracy measures of cognitive performance across the lifespan, administering the same tasks to children, younger adults, and older adults. Six processing speed tasks (Response Time (RT) tasks, 8 conditions) and two working memory tasks scored in terms of the number of correct responses (Working Memory (WM)—verbal and visuo-spatial, 6 conditions), as well as control tasks, were administered to over 500 individuals distributed across the three age periods. The main questions were whether age differences in IIV would vary throughout the lifespan according (i) to the type of measure used (RTs vs. accuracy); and (ii) to task complexity. The objective of this paper was to present the general experimental design and to provide an essentially descriptive picture of the results. For all experimental tasks, IIV was estimated using intra-individual standard deviation (iSDr), controlling for the individual level (mean) of performance and for potential practice effects. As concerns RTs, and in conformity with a majority of the literature, younger adults were less variable than both children and older adults, and the young children were often the most variable. In contrast, IIV in the WM accuracy scores pointed to different age trends—age effects were either not observed or, when found, they indicated that younger adults were the more variable group. Overall, the findings suggest that IIV provides complementary information to that based on a mean performance, and that the relation of IIV to cognitive development depends on the type of measure used.

## 1. Introduction

Almost all research in cognitive psychology, including cognitive developmental psychology, has focused on mean performance. Yet, individual variability should not be neglected, whether inter- or intra-individual. Many authors have proposed to consider that individual variability should be taken into consideration when proposing general laws of behavior (e.g., Reference [1]). That is, general psychology should be able to integrate the bulk of individual variability, just as much as differential psychology should also lean on experimental manipulations (e.g., Reference [2]). Several types of individual variabilities should be distinguished (e.g., Reference [3]). Inter-individual variability has been often acknowledged, for instance when comparing groups or individuals from an applied perspective, or when standard deviations are reported. Note, however, that standard deviations only provide information about quantitative differences among individuals, as if they differed solely in how distant from their group mean they are; they do not provide any indication on qualitative differences.

Intra-individual variability (IIV) can be further distinguished as a function of the time period considered: across long-term periods (developmental change), across trials within tasks (inconsistency), and across tasks at a given point in time (dispersion). The present study focused on short-term within-task intra-individual variability, that is, on inconsistency or fluctuations across trials [4,5,6], and examined whether age differences (i) are observed throughout the lifespan; and (ii) vary across tasks. It has been argued already years ago that within-task variability might represent better the entire performance than a measure of central tendency alone [3,7,8,9]. A new gain in interest has recently emerged again, backed up by empirical data. The interest for a change in amplitude of IIV with age has primarily been demonstrated in the cognitive aging domain [4,10] and is now also burgeoning in cognitive developmental research in children [6,11,12,13,14].

Note that the label “inconsistency”, the focus of the present study, attached to within-task intra-individual variability shows that IIV usually bears a negative connotation, as a cursory review of literature attests. IIV has indeed been shown to be a predictor of cognitive dysfunction. A first set of studies by Hultsch and collaborators [15] has compared three groups of older adults (mildly demented, physically ill with arthritis, and healthy adults) and observed that the demented persons presented a much larger IIV in latency and accuracy of cognitive performance. Moreover, IIV was uniquely predictive of neurological status, independent of the level of performance. IIV is also presented as a useful marker of cognitive functioning in children with learning disabilities, such as children with attention-deficit hyperactivity disorder (ADHD), as compared to children with typical development [11,12].

With respect to age, IIV has been observed to be larger in children and older adults [4,16,17,18,19,20,21,22,23,24,25,26,27]. Cross-sectional studies showed a developmental trend across the lifespan similar to that of the mean level of performance: A typical U-shaped curve with a decrease in IIV in response time (RT) from childhood to young adulthood followed by an increase throughout adulthood relatively early on [6,22,28,29,30]. Older adults were found to be more inconsistent than younger ones on RT measures in simple tasks, and baseline differences in IIV were shown to be associated with the level of performance. A few aging studies were longitudinal, having followed the same individuals across several years [19,27]. They showed an increase in IIV from adulthood to late adulthood with an acceleration beyond the age of 70 [24]. There are, however, some discrepancies in the literature, some studies reporting no age effect in IIV [31,32].

IIV is thus considered as an important source of information in addition to the mean performance in both aging and child development [33,34]. Yet, although steadily increasing, the empirical evidence is still far from sufficient to demonstrate the usefulness of adding a measure of variability to that of the mean level, and its specificity. Moreover, a number of methodological and theoretical issues and questions are pending, some of which will be examined in the present paper. First, it is not always the case that IIV is larger in older adults, or in children, once the mean RT is controlled for (e.g., Reference [35]). Intra-individual standard deviations are often used to study IIV; however it is known that, even though means and standard deviations are considered to be statistically independent, they tend to vary together [36]. Larger mean reaction times, as observed in children and older adults for instance, will therefore tend to be associated with larger standard deviations. Hence, it is important to control for the level of performance when estimating IIV. Second, although a similar (and inverse) trend has been observed in childhood and older adulthood as concerns mean performance, few studies have analyzed IIV across the entire lifespan, from childhood to advanced old age, using the same tasks in the different age groups (but see [6,22,30]). Third, nearly all studies on IIV have relied on RT tasks, while few studies have focused on correct responses (accuracy performance scores). Yet, a number of cognitive processes, and thus tasks, need to be assessed taking into account both level of accuracy and correct responses. This is the case of memory tasks, including complex working memory (WM) span tasks, in which accuracy scores (such as span scores) are more generally considered. The use of other scores and non RT tasks might explain why some authors did not observe age differences in IIV (e.g., Reference [31,32]).

Furthermore, the meaning of a large IIV is not very clear from a theoretical point of view. Larger IIV is often associated with vulnerability or impairment, as mentioned above with respect to the use of the term inconsistency. However, in the child developmental domain, IIV is not specifically linked to learning disabilities, but simply with younger age; young children show larger IIV than older children [6,12]. It is noteworthy to add that IIV has also been addressed from a different perspective as concerns development in children. Siegler [13] or van Geert and van Dijk [37] often emphasized a larger variability in younger children, and considered it to index the development of multiple strategies or/and to reflect the upcoming of a stage transition. Not only can IIV be considered as a “prominent feature in child development,” but also it “is an important characteristic of self-organization in development” as viewed already by dynamic systems theorists in the early 1990’s ([38], p. 53). In this case, IIV would thus reflect a developmental progress.

The question of whether large IIV is dysfunctional or adaptive (pointing to resilience) is still being presently discussed in the aging domain [39,40]. Also, and in spite of the quantitative similarity in IIV trends in childhood and in aging, different processes probably account for IIV changes [41,42]. During childhood, among several factors, the maturation of white matter such as myelination [43] has been suggested to be linked to the decrease in IIV. For older adults, IIV might reflect structural and functional changes in frontal lobe functions, as well as in dopaminergic neuromodulation [44], in the quality/integrity of white matter [43,45,46,47,48] or in neuromodulatory efficiency [19,22]. In particular, the quality of white matter seems to present a stronger relation with IIV than with the mean level of performance, a result that has been interpreted as reflecting a less stable communication between cerebral regions [45,47,49].

The present study pursued the following objectives: (i) Examine age trends in inconsistency throughout the lifespan by administering the same tasks to children, younger adults and older adults; (ii) Compare IIV in both RT and in working memory (WM) tasks assessing accuracy; (iii) Compare tasks of varying complexity, to determine whether IIV would be larger in more complex tasks and assessing for possible interactions of age and complexity.

Several tasks were administered to all individuals: six RT tasks varying in complexity, from a simple RT task to Choice RT tasks (Lines comparison, Cross-square), complex processing speed tasks (Digit Symbol and Comparison of letters), an interference task (Stroop), as well as two WM span tasks (Reading Span and Visuo-spatial WM) also varying in complexity. All the tasks were administered in an identical manner to children, younger adults and older ones. IIV was examined in each task, using an intra-individual standard deviation, residualized for the individual level of performance (iSDr, see method). The use of residualized scores before computing an iSD is in line with the suggestion by Hultsch and coll. (e.g., Reference [50]).

Our expectation, based on the extant literature, was that both children and older adults would exhibit larger IIV than younger adults in the RT tasks [6,10,23,51,52,53]. We expected also more variability in the more difficult condition (complex RT tasks), and an interaction with age, the difference being larger in children and older adults. The question of whether the same trend would be observed for IIV in the two WM tasks was left open. There are several reasons why the age trend of IIV might differ between the two types of tasks, such as the discordant results obtained with the two types of scores [31], the range of possible responses—smaller for the WM tasks than the RT tasks [54]—and the different implications of a larger IIV. In RT tasks, larger IIV has been observed in those individuals considered to present a lower performance, that is, those with longer RTs. In the case of a WM task, it is known that children and older adults present lower scores than younger adults. If IIV was indeed indicative of lower cognitive functioning, it should be associated with lower scores in contrast with what is observed in RT tasks.

## 2. Materials and Methods

### 2.1. Participants

Two hundred and one children (age range = 9–12 years, tested within 2 months of their birthday), 137 younger adults (age range = 19–33), and 219 older adults (age range = 59–89) participated in the study^1^. Children were recruited from primary schools in the city of Geneva. The younger adults were undergraduate students at the University of Geneva participating for course credit. The older adults were volunteers recruited from the community, either from the University of the Third Age of Geneva, or through newspaper and association advertisements for pensioners. None of the older adults, as shown by a large battery of tasks administered (e.g., Reference [55]), presented an incipient dementia. Only participants who spoke French either as their first language or fluently were included.

The French version of the Mill-Hill vocabulary scale [56] was administered to the younger and older adults. A one-way ANOVA on vocabulary scores in the two adult groups indicated a main effect of age, *F*(1, 351) = 45.489, *p* < 0.001, η_p_^2^ = 0.12; as frequently observed in aging studies for abilities related to crystallized intelligence (e.g., Reference [57]), younger adults had a lower vocabulary score than older adults (*p* < 0.05). Additionally, the Raven’s Progressive matrices task (Raven, 1938) was administered to all participants. A one-way ANOVA on correct responses indicated a main effect of age, *F*(2, 552) = 197.33, *p* < 0.001, η_p_^2^ = 0.42; younger adults had better performances than children and older adults (*p* < 0.001). Children and older adults did not differ significantly.

Descriptive statistics for the demographic variables of the participants are provided in Table 1 (see also Table A1 in Appendix A).

### 2.2. Materials and Procedure

All participants were administered the same tasks, in a quiet room either at school for children or in our laboratory for adults, during two or three sessions, at most one week apart. All tasks were individually administered on a Dell computer using E-Prime [58] in the same order for all participants.

Latency scores. Six tasks (9 experimental conditions) were used to assess reaction times (RTs): one simple reaction time task (SRT), two choice reaction time tasks (line comparison—LI, and cross-square—CS), two processing speed tasks (digit symbol—DI, and letter comparison—LC, 2 conditions, 6 vs. 9 letters) and one interference task^2^ (Stroop color-word, 2 conditions, neutral vs. incongruent).

Accuracy scores. Two working memory (WM) tasks were used. In an adaptive version of the Reading span task [59,60], participants had to judge series of sentences while memorizing the last word; at the end of a series, they had to retrieve all the words. In the Matrices task, a grid of 5 × 5 cells was presented on a touch screen, with either a certain number of cells colored (spatial condition) or words contained in certain cells (spatio-verbal condition); participants had to point at the previously colored cells onto an empty matrix (Position score), or orally retrieve the word and simultaneously indicate its position onto the screen (Word-position score). For these two tasks, increasing series of sentences or number of positions (position or word-position) were first presented in a preliminary phase to assess the span level of each individual^3^. In order to make it possible to compute an intra-individual standard deviation, ten trials were then presented for each of two complexity conditions: at the individual’s span level, and at span + 1 level^4^. Scores consisted of the number of correctly recalled items for each condition.

Brief descriptions of all these tasks are provided in Table 2; for more details on the procedure see [1]. For all tasks, only the correct responses contributed to the score.

Note: For the Stroop task, the three conditions were randomly distributed within each block. Only the conditions Neutral (colored geometrical signs) and Incongruent (colored word colored in a different color, e.g., red colored in blue) were used in the analyses. The Reading span was administered twice, with 10 trials per condition; the two sessions were pooled in the present analyses.

### 2.3. Statistical Analyses

Analyses were conducted on latency scores in the RT tasks and on the accuracy scores of the WM tasks. For all tasks, we conducted analyses on the mean performance (iM) and on the intra-individual standard deviation (iSDr)^5^. For the latter, scores were first residualized (standardized residual scores) for the mean level of the individual (iM), as well as for the order of trials and blocks, to control for possible practice effects. In the WM tasks, analyses were conducted on the number of correctly recalled items for each trial (words in the Reading span tasks, positions, and words/position^6^ in the Matrices conditions). Then, intra-individual standard deviation (iSDr) were computed on those scores residualized for iM, order of trials and of blocks.

## 3. Results

### 3.1. Reaction Times Tasks

Descriptive statistics for the different RT tasks used are presented in Table 3; Table A2 reports the statistics for the five age groups. The data were submitted to one-way ANOVAs, comparing the age groups, by condition. Thresholds of significance were corrected for multiple comparisons (Bonferroni correction for a threshold of 0.05; *p* < 0.004).

#### 3.1.1. Intra-Individual Mean (iM)

- Simple RT task. The main effect of age was significant, *F*(2, 554) = 74.78, *p* < 0.001, η^2^ = 0.21. Children were significantly slower than older adults (*p* < 0.001), themselves being significantly slower than younger adults (*p* < 0.001). Furthermore, children were significantly slower than younger adults (*p* < 0.001).
- Choice RT tasks. For the LI and CC tasks, the main effect of age was significant, *F*(2, 554) = 257.23, *p* < 0.001, η^2^ = 0.48 and *F*(2, 554) = 175.42, *p* < 0.001, η^2^ = 0.39 respectively. For both tasks, children were significantly slower than older adults (*p* < 0.001), themselves being significantly slower than younger adults (*p* < 0.001). Furthermore, children were significantly slower than younger adults (*p* < 0.001).
- Processing speed tasks. For the DI task, the main effect of age was significant, *F*(2, 554) = 170.97, *p* < 0.001, η^2^ = 0.38. Children were significantly slower than younger adults (*p* < 0.001), themselves significantly faster than older adults (*p* < 0.001). Children did not differ significantly from older adults. For the LC task, the main effect of age was significant, *F*(2, 552) = 161.99, *p* < 0.001, η^2^ = 0.37. Children were significantly slower than older adults (*p* < 0.001), the latter being significantly slower than younger adults (*p* < 0.001). Furthermore, children were significantly slower than younger adults (*p* < 0.001). A main effect of condition was also observed, *F*(1, 552) = 2067.01, *p* < 0.001, η^2^ = 0.79. Participants were significantly faster in the 6 letters condition than in the 9 letters condition. Finally, the age group x condition interaction was significant, *F*(2, 552) = 26.54, *p* < 0.001, η^2^ = 0.09. Post-hoc comparisons revealed that the age group effect was significant for all conditions (all *p*s < 0.001). Moreover, the main effect of condition was significant for all age groups (all *p*s < 0.001); the interaction reflects a more pronounced effect for children and older adults than for younger adults.
- Stroop task. The main effect of age was significant, *F*(2, 548) = 164.56, *p* < 0.001, η^2^ = 0.38. Children were significantly slower than older adults (*p* < 0.001), themselves being significantly slower than younger adults (*p* < 0.001). Furthermore, children were significantly slower than younger adults (*p* < 0.001). A main effect of condition was also observed *F*(1, 548) = 2721.60, *p* < 0.001, η^2^ = 0.83. Participants were significantly faster in the neutral condition compared to incongruent condition. Finally, the age group x condition interaction was significant, *F*(2, 548) = 44.96, *p* < 0.001, η^2^ = 0.14. Post-hoc comparisons revealed that the age group effect was significant for all conditions (all *p*s < 0.001). The main effect of condition was significant for all age groups (all *p*s < 0.001); this effect seemed more pronounced for children and older adults than for the younger adults, as shown by a significant interaction effect.

In sum, in all RT tasks, children and older adults were slower than the younger adults; children were also slower than older adults except in DI (see also footnote 7).

#### 3.1.2. Intra-Individual Standard Deviation of Residual Scores (iSDr)

- Simple RT task. The main effect of age was significant *F*(2, 554) = 99.18, *p* < 0.001, η^2^ = 0.26. Children were significantly more variable than older adults (*p* < 0.001), who were significantly more variable than younger adults (*p* < 0.001). Furthermore, children were significantly more variable than younger adults (*p* < 0.001).
- Choice RT tasks. For the LI and CC task, the main effect of age was significant, *F*(2, 554) = 180.83, *p* < 0.001, η^2^ = 0.40 and *F*(2, 554) = 190.26, *p* < 0.001, η^2^ = 0.41 respectively. Children were significantly more variable than older adults (*p* < 0.001), themselves being significantly more variable than younger adults (*p* < 0.001). Furthermore, children were significantly more variable than younger adults (*p* < 0.001).
- Processing speed tasks. For the DI task, the main effect of age was significant, *F*(2, 554) = 215.70, *p* < 0.001, η^2^ = 0.44. Children were significantly more variable than older adults (*p* < 0.001), themselves being significantly more variable than younger adults (*p* < 0.001). Furthermore, children were significantly more variable than younger adults (*p* < 0.001). For the LC task, the main effect of age was significant, *F*(2, 552) = 211.35, *p* < 0.001, η^2^ = 0.43. Children were significantly more variable than older adults (*p* < 0.001), themselves being significantly more variable than younger adults (*p* < 0.001). Furthermore, children were significantly more variable than younger adults (*p* < 0.001). A main effect of condition was also obtained, *F*(1, 552) = 29.06, *p* < 0.001, η^2^ = 0.05. Participants were significantly less variable in the 6 letters condition than in the 9 letters condition. Finally, the age group x condition interaction was significant, *F*(2, 552) = 5.42, *p* < 0.005, η^2^ = 0.02. Post-hoc comparisons revealed that the age group effect was significant for all conditions (all *p*s < 0.001). Moreover the main effect of condition was significant for younger adults and older adults (all *p*s < 0.001), but not for children.
- Stroop task. The main effect of age was significant, *F*(2, 548) = 251.85, *p* < 0.001, η^2^ = 0.48. Children were significantly more variable than older adults (*p* < 0.001), themselves significantly more variable than younger adults (*p* < 0.001). Furthermore, children were significantly more variable than younger adults (*p* < 0.001). A main effect of condition was also significant, *F*(1, 548) = 32.23, *p* < 0.001, η^2^ = 0.06. Participants were significantly less variable in the neutral condition than in the incongruent condition. Finally, the age group x condition interaction was significant, *F*(2, 548) = 32.23, *p* < 0.001, η^2^ = 0.06. Post-hoc comparisons revealed that the age group effect was significant for all conditions (all *p*s < 0.001). Moreover, the main effect of condition was significant for all age groups (all *p*s < 0.001), this effect seemed more pronounced for older adults, leading to a significant age group x condition interaction.

To sum up^7^, whichever the task, children were slower and more variable than older adults; the latter were slower and more variable than younger adults. It should be noted that for the DI task, children and older adults did not differ significantly in their intra-individual means (iM). Also, a condition effect showed, leading to larger RTs and increased variability: Participants were slower and more variable in the 9-letter LC condition than in the 6-letter one, as well as in the ST Interference condition compared to the Neutral one. Note, however, that children were not more variable in the 9-letter condition than in the 6-letter one.

### 3.2. Working Memory Tasks

Descriptive statistics for the different scores used are presented in Table 4. Table A2 provides descriptive data for the initial phases of the WM tasks (assessment of span level). The data of each task were submitted to 3 × 2 repeated-measures ANOVAs with age group (children, younger adults, older adults) as between-subject factor and list length (*n* and *n* + 1) as within-subject factor. The decision criterion was adapted using the Bonferroni procedure (*p* < 0.004 corresponding to a 0.05 threshold).

#### 3.2.1. Intra-Individual Mean (iM)

- Reading span task. The analysis of the mean number of correctly recalled words indicated a main effect of age group, *F*(2, 511) = 57.31, *p* < 0.001, η_p_^2^ = 0.18. Children recalled significantly fewer words than older adults (*p* < 0.001), themselves recalling significantly fewer words than younger adults (*p* < 0.001). Furthermore, children recalled significantly less words than younger adults (*p* < 0.001). A main effect of list length was also found, *F*(1, 511) = 504.61, *p* < 0.001, η_p_^2^ = 0.50. Participants recalled significantly fewer words for the n list than for the *n* + 1 list. Finally, the age group x list length interaction was significant, *F*(2, 511) = 9.23, *p* < 0.001, η_p_^2^ = 0.04. Post-hoc comparisons revealed that the age effect was significant for both n and *n* + 1 lists (*p* < 0.001). Moreover, the list length effect was significant for all age groups (*p* < 0.001); this effect seemed more pronounced for younger adults than for children and older adults, leading to a significant age group x list length interaction.
- Matrices task—Simple positions. The main effect of age group was significant, *F*(2, 550) = 138.53, *p* < 0.001, η_p_^2^ = 0.34. Children recalled significantly fewer positions than younger adults (*p* < 0.001). Older adults recalled significantly fewer positions than younger adults (*p* < 0.001). Children did not differ significantly from older adults. A main effect of list length was also obtained, *F*(1, 550) = 992.25, *p* < 0.001, η_p_^2^ = 0.64. Participants recalled significantly fewer positions for the *n* list than for the *n* + 1 list. Finally, the age group x list length interaction was significant, *F*(2, 550) = 15.31, *p* < 0.001, η_p_^2^ = 0.05. Post-hoc comparisons revealed that the main age effect was significant for both *n* and *n* + 1 lists. Children recalled significantly fewer positions than younger adults and older adults recalled significantly fewer positions than younger adults; *p* < 0.001. Moreover, the list length effect was significant for all age groups (*p* < 0.001); this effect seemed more pronounced for younger adults (cf. significant age x condition interaction).
- Matrices task—word-position associations. Results indicated only a main effect of age, *F*(2, 548) = 121.68, *p* < 0.000, η_p_^2^ = 0.31. Children recalled significantly fewer associations than older adults (*p* < 0.006), themselves recalling significantly fewer associations than younger adults (*p* < 0.000). Furthermore, children recalled significantly fewer associations than younger adults (*p* < 0.000). The effects of list length and its interaction with age were not significant.

To sum up, in all tasks, children and older adults recalled fewer items (words in the Reading span task, positions and word-position associations in the Matrices task) than younger adults. Children recalled fewer words and fewer word-positions associations than older adults. Interestingly, both groups did not differ in the number of positions retrieved, except as concerns positions in the Matrices task.

#### 3.2.2. Intra-Individual Standard Deviation of Residual Scores (iSDr)

- Reading span task. Results indicated only a main effect of list length, *F*(1, 511) = 23.57, *p* < 0.001, η_p_^2^ = 0.04. Participants were significantly less variable for the *n* list than for the *n* + 1 list. Neither the effects of age group nor its interaction with list length were significant.
- Matrices task—Simple positions. Neither effects of age and list length nor their interactions were significant.
- Matrices Task—word-position associations. Results indicated only a main effect of age, *F*(2, 548) = 29.47, *p* < 0.001, η_p_^2^ = 0.10. Children were significantly less variable than younger adults (*p* < 0.001). Older adults were significantly less variable than younger adults (*p* < 0.001). Children did not differ significantly from older adults.

To sum up^8^, as concerns iSDr, only word-position associations in the matrices task showed an age effect, with younger adults being more variable than children and older adults.

## 4. Discussion

As discussed in the introduction, intra-individual variability (IIV) can provide crucial information, at least when RTs are examined, on both normative development/aging and processing impairment, beyond the information associated with the mean level (e.g., Reference [61]). The novelty of the present study was to examine IIV across the lifespan, in particular within-task fluctuations (inconsistency), in both latencies, -RTs-, and in accuracy -WM- tasks that varied with complexity. An additional contribution was to use the same tasks in all the age groups. We analyzed six RT tasks (8 conditions) using response times, and two WM tasks (6 conditions) to children, younger and older adults using an accuracy score based on the number of items recalled by trial. We were also interested in determining whether IIV would vary with complexity. The RT tasks ranged from a simple RT task in which one had simply to detect a target (SRT), to simple choice RT tasks (LI and CS), to more difficult processing speed tasks (DI and LC) in which two longer series of items had to be compared. A Stroop task was used, comparing a neutral and an incongruent condition, considered as more difficult. Finally, two WM tasks were used, which differed from the RT tasks not only because an accuracy score was used, but also because they are altogether much more complex tasks to process; moreover, two levels of complexity were presented for each task. It was expected that IIV would increase with complexity, in the two groups of tasks. 

As expected, and in line with previous studies, results for both groups of tasks showed age-related differences in the mean level of performance between children, as well as between younger adults and older adults, replicating observations made in development [41], or in aging [62,63,64] or in studies conducted across the lifespan [6,22,30,65]. Younger adults had shorter RTs (they processed information faster) and recalled a larger number of correct items in the WM tasks than children, on the one hand, and than older adults, on the other hand. Such a pattern of results confirms that (i) processing speed increases in efficiency from childhood to adulthood and then decreases again in older adults; older adults were nevertheless still faster than children; (ii) WM performance increases from children to younger adults and then decreases again in older adults. Age differences were, however, less marked in the WM tasks, depending on the condition; this was particularly the case when age groups were analyzed more finely (distinguishing younger and older children, on the one hand, and young-old and old-old adults, on the other hand—see Footnotes 6 and 7). Taken together, these results are consistent with the well-known mean age trends across the lifespan.

When intra-individual standard deviations (iSDrs) were considered, once the individual mean performance controlled for, age differences varied depending on the measure considered (RTs vs. accuracies). In line with a majority of previous studies [6,21,62], the picture remained more or less similar as concerns the RTs: Younger adults were the least variable and children were the most variable. The additional analyses in finer age groups showed that younger (9–10 years of age) and older children (11–12 years of age) also differed significantly; a difference was also observed between the young-old and old-old adults, the latter being more variable in all the tasks, except in LC. IIV in the WM tasks presented a very different pattern with respect to age differences. Even though most differences were not significant, the highest iSDrs were obtained in younger and older adults, and the lowest in children. The only significant age effect was obtained in the Matrices task, for the word-positions associations, that is, when the information to be retrieved is rather complex. In that condition, children and older adults did not differ, but younger adults differed from both age groups. The additional analyses in five age groups showed no further age difference between the two children groups, or between the two older adults groups. Overall, one can thus conclude that there was no age effect in most conditions of the WM tasks, and when one was observed, it pointed to a larger variability in younger adults than in the other groups.

The results obtained in the RT tasks are compatible with the hypothesis often adopted in the literature [35] that IIV reflects reflect failures in attentional control or attentional lapses in children and older adults. Furthermore, these results were obtained independently from the IIV index used (raw intra-individual standard deviation—iSD-, or iSDr computed on the RTs residualized for age group as presented here). IIV in WM accuracy scores offered a different picture. Very interestingly, the age differences tended to be reversed, although not significantly so in all conditions. Younger adults were not less variable than the two other age groups; they were even significantly more variable in the most complex condition of the Matrices task (word-position associations for both span levels). The iSDrs in younger children were the smallest, for all conditions, even if not significantly so. Thus, results clearly showed that children and older adults were not more variable than younger adults. We mentioned in the introduction that other studies using an accuracy score and analyzing IIV also failed to support a larger IIV in memory [15] or in WM performance [31] not only in older adults but also in children.

If this was the case that IIV in general relates to attentional control and to brain development, the age differences in WM accuracy performance would be similar to those obtained in RTs, all the more so as WM is supposed to call for more attentional or executive processes than speed measures. IIV in accuracy might also reflect attentional lapses (i.e., irregularities in attentional control) like IIV in RTs, but it might just as well point to a greater diversity in the strategies used; it is known that the repertoire of strategies increases with development as Siegler [13] observed in children learning arithmetical skills. On this basis, one would indeed expect that young adults be more variable. One could also adopt a slightly different perspective, and consider that IIV increases with the proximity to a developmental transition [38]. We will come back later to the possible role of strategies.

The failure to observe a larger IIV in WM performance in children and older adults could find a number of other explanations, among which essentially the task characteristics. First, the number of trials, which is typically low in WM tasks, might not be sufficient to obtain a reliable score. In contrast to simple RT tasks, it is indeed difficult to administer a WM task with a very large number of complex trials, in particular because trials should preferably not be repeated, but also in order not to fatigue participants and make the task too complex to be completed, leading to discouragement. Therefore, tasks focusing on accuracy often do not use a sufficient number of trials to provide a reliable indicator of IIV. In the present study, iSDr was computed across ten or twenty trials by condition for the WM tasks; this is certainly sufficient, but nevertheless less than in the RT tasks (from 60 to 144 trials by condition). Second, a factor that could play a role in explaining the present results might be the task complexity: It is possible that IIV in accuracy of WM performance is more sensitive to the task complexity (e.g., larger in *n* + 1 than in n trials- rather than to age differences per se). Yet, as expected, complexity also played a role in RT tasks: iSDrs were larger in the more difficult speed tasks and this difference was significant in all groups when it could be tested, that is, in the LC and the Stroop tasks. Thus, complexity might be relevant but is not sufficient to account for the difference between IIV in RT and WM tasks. Third, accuracy scores might lack sensitivity. The grain of variation is, indeed, coarser. In WM tasks, scores typically range from zero to six or seven as a maximum (the mean performance of younger adults was around 3, except for the Matrices-Positions score which was around 6); one item more or one less to be memorized represents therefore a large difference. Moreover, the difficulty of the task was adapted to the individual’s span, probably leading to fewer fluctuations. In contrast, there is a continuous and large range of potential variations in RT tasks (i.e., mean of around 300–400 ms in young adults in the simpler RT tasks, and greater than 1000 ms in the more complex conditions). Fourth, in RT tasks, the range of possible responses has only a lower bound but not really an upper bound. Therefore, considering the relationship between the individual mean performance and IIV [54], the longer the RTs, the larger the magnitude of the variability may be. In accuracy tasks, the entire interval of responses is delimited, between 0 and the upper limit of the WM trial administered. Consequently, the variability is not “observable” when the scores are too low (floor effect) or too high (ceiling effect). This is incidentally one of the reasons why the length of the series administered was adapted to the individual’s span.

It could finally be argued that the difference between the two types of tasks is linked to a speed-accuracy trade-off, taking place essentially in the RT tasks but probably not in the WM ones. Older adults might be more concerned than younger adults by maintaining accuracy rather than speed, and control their response to the expense of a heightened variability in speed. However, there were no age differences in the number of errors in the RT tasks and the number of errors was very small (somewhat higher in the Interference condition of the Stroop task). Also, this speed-accuracy trade-off hypothesis is rather unlikely, as it would lead to a same conclusion as regards children. Although, at least to our knowledge, there are no empirical studies on this topic, it does not seem plausible that children would deliberately slow down in some trials in order to maintain a high rate of accuracy, and more so than younger adults.

A different, and more global, argument could be of a more statistical nature: Variability might simply be higher when mean scores are higher. Therefore young adults would show less intra-individual variability in the RT tasks, and more variability in the WM tasks, as in the present study. This is precisely why we relied on residualized intra-individual standard deviations, controlling for mean score. Perhaps this correction is not sufficient. Note, however, that this argument applies to both types of tasks, and that, usually, authors working on intra-individual variability in RT tasks attribute a psychological rather than a statistical meaning to a larger variability in older adults. Moreover, this argument would not in turn account for the fact that the age group has much less effect in the WM tasks even though the mean scores are clearly sensitive to age. Results clearly are not symmetrical between the two types of tasks: They clearly show that the tasks differ but not that intra-individual variability is significantly larger in the young adults. We therefore still need an additional explanation to account for the difference.

From a behavioral point of view, and as concerns age differences, it may be that IIV in performance in WM tasks is less informative than IIV in RTs or more interestingly that it points to different underlying processes. For instance, as we discussed above, IIV could reflect a change in the strategies used, more so in the WM tasks where they are more useful. Examples of strategies are attempting to chunk the last words in the Reading span task, or finding a mnemonic to associate word and position in the Matrices task. Of course, strategies can also be used in the RT tasks (for instance memorizing the reference matrix in the DI task, or comparing subgroups of letters in the LC task); they are useful for responding faster but not necessarily for achieving higher accuracy in a task that remains relatively easy to process. Strategies are probably more numerous in adults, but might differ in their applicability across items; moreover, switching strategy might contribute to maintaining a higher performance in the case of a temporary failure in other processes; in both cases, their use would lead to larger intra-individual variability. Variability might therefore prove more adaptive in WM tasks than in RT tasks and reflect the underlying dynamics [39]. It is not possible, however to test this hypothesis in the present version of our WM tasks because we did not question the participants on how they maintained and retrieved items. It is also possible that different brain mechanisms underlie IIV in RT tasks, on the one hand, and IIV in accuracy in complex WM tasks, on the other hand. As mentioned in the introduction, a larger IIV in RTs has been shown to be associated with structural and functional brain changes; however, to date, there is very little evidence of an association between IIV in WM and brain processes. In a recent study on older adults using Diffusion Tensor Imaging, IIV in RTs was shown to be correlated with fractional anisotropy, and more strongly so than the mean RT score; no such correlation was observed for IIV in verbal WM IIV [46]. A final argument supporting an essential difference between RT and WM tasks has been brought by another study focusing this time on dispersion in the same sample, that is on intra-individual variability across tasks rather than across trials [66]. In this study, young adults were less variable in their mean level across the RT tasks than both children and older adults; they were, however, more variable across the conditions of the WM tasks (the same two tasks as in the present study).

To clarify those issues further, future studies should make the effort to replicate the present results also by (i) using different tasks while also combining RT and accuracy scores; and (ii) analyze within a same WM task accuracy and RTs (note that response time is not really relevant in a complex WM task such as the Reading span task). Perhaps, also, it would be worthwhile to examine more contiguous age groups than we did in the present study.

## 5. Conclusions

To conclude, our results suggest that inconsistency—IIV across trials—is informative of age-related differences across the lifespan when RTs are considered but less so when performance in WM task is examined. This issue does not preclude the interest to study IIV in WM accuracy scores, to assess first whether it varies across different tasks, and second whether it remains a predictor of individual differences (beyond age differences) in cognitive functioning. The present study clearly demonstrates that IIV does not behave similarly in these two types of tasks, even though they were administered to the same individuals. Future studies should pursue the question raised here of whether different processes are involved in IIV, depending on the type of task and of score used.

## Figures and Tables

**Table 1:** Participants’ Characteristics by Age Group.

	Age Groups					
	Children		Young Adults		Older Adults	
N	201		137		219	
female	92		117		165	
	M	SD	M	SD	M	SD
Age	10.50	1.12	21.71	2.53	70.10	6.78
Vocabulary Score	_	_	34.67	3.25	37.73	4.64
Raven	36.84	8.21	52.15	4.91	36.47	9.12

Note: M: Group’s mean; SD: Group’s standard deviations.

**Table 2:** Descriptions and characteristics of tasks used in this study.

	Category	Task	Instructions	Condition	Trial
Latency score	Simple reaction Time	SRT	To detect as quickly as possible when a stimulus appeared on the screen	-	120
	Choice reaction time	LI	To detect on which of two sides the longest of two lines was located	-	120
		CS	To detect on which of two sides one of the six crosses changed into a square	-	120
	Processing speed	LC	to decide whether two series of letters were identical or not	6 or 9 letters	60
		DI	to determine whether a number–symbol pair was similar to a reference matrix	-	144
	Interference	ST	To name the color in which words or signs were written	Neutral Congruent Incongruent	144 144 144
Accuracy score	Working memory task	Rspan	To memorize and recall words	Span level Span + 1 level	20 20
		Matrices	To memorize and recall positions	Span level Span + 1 level	10 10
			To memorize and recall word- positions associations	Span + 1 level Span + 2 level	10 10

Note: SRT: Simple reaction time task. LI: Line comparison task; CS: Cross-square task; LC: Letter comparison task; DI: Digit symbol task; ST: Stroop task; Rspan: Reading span task.

**Table 3:** Reaction time tasks: Means (iM) and intra-individual standard deviation of residual scores (iSDr) by Age group.

			Age Groups					
			Children		Young Adults		Older Adults	
			M	SD	M	SD	M	SD
iM	SRT		360.03	68.78	272.89	39.16	333.50	73.41
	LI		565.38	101.31	372.38	43.52	457.66	70.46
	CS		495.57	105.41	325.69	39.26	431.16	76.75
	DI		1737.32	447.94	1040.56	221.47	1668.96	352.86
	LC	6 letters	3626.06	1236.06	1838.17	423.31	2971.24	799.48
		9 letters	5092.16	1550.37	2798.36	654.47	4316.61	1114.48
	ST	Neutral	842.21	146.76	594.77	75.85	724.31	110.05
		Incongruent	1019.96	184.77	720.89	102.30	927.75	166.67
iSDr	SRT		11.38	3.04	7.17	2.15	9.23	2.75
	LI		12.24	3.48	6.42	1.98	8.58	2.72
	CS		12.27	3.94	5.93	1.78	8.86	2.50
	DI		12.04	3.55	5.69	1.91	9.23	2.35
	LC	6 letters	12.56	5.22	5.03	1.67	8.17	2.86
		9 letters	12.64	4.11	5.83	1.81	8.88	2.56
	ST	Neutral	12.97	3.85	6.16	1.91	7.71	2.81
		Incongruent	12.40	2.95	7.16	1.92	8.83	2.55

Note: SRT: Simple reaction time task; LI: Line comparison task; CS: Cross-square task; LC: Letter comparison task; DI: Digit symbol task; ST: Stroop task; iM: intra-individual mean; iSDr: standard deviation of residual scores; M: Group’s mean; SD: Group’s standard deviations.

**Table 4:** Accuracy tasks: Means (iM) and intra-individual standard deviation of residual scores (iSDr) by Age group.

			Age Groups					
			Children		Young Adults		Older Adults	
			M	SD	M	SD	M	SD
iM	Reading span	List length *n*	2.08	0.54	2.83	0.76	2.46	0.76
		List length *n* + 1	2.35	0.58	3.27	0.69	2.82	0.77
	Matrices tasks—Positions	List length *n*	3.35	1.33	5.32	1.41	3.30	1.09
		List length *n* + 1	4.03	1.48	6.14	1.53	3.82	1.10
	Matrices tasks—associations	List length *n* + 1	2.25	0.68	3.24	0.63	2.51	0.65
		List length *n* + 2	2.25	0.70	3.29	0.75	2.37	0.73
iSDr	Reading span	List length *n*	8.43	3.31	9.20	4.84	9.84	4.56
		List length *n* + 1	9.59	2.23	10.14	2.90	10.08	2.75
	Matrices tasks—Positions	List length *n*	9.70	4.76	8.49	6.03	9.12	4.98
		List length *n* + 1	9.38	3.72	8.92	5.03	10.23	4.31
	Matrices tasks—associations	List length *n* + 1	9.50	2.65	11.28	4.40	9.77	3.34
		List length *n* + 2	9.35	2.51	11.49	3.42	9.98	2.66

Note: iM: intra-individual mean; iSDr: standard deviation of residual scores; M: Group’s mean; SD: Group’s standard deviations.

## Footnotes

1 For the Reading span task, a memory score was computed only when the participants responded correctly for at least 85% of the sentences. This precaution was adopted to insure that participants did process the sentence while retaining the last word, that is, that the task was a dual one. The participants who made more errors when judging the sentence were attributed a missing score. Note that the sentences were simple. Overall, forty-one participants (essentially children) were discarded from the analyses because they did not reach the 85% accuracy criterion on the judgment task.

2 The Stroop task was administered twice, at one week intervals, to split the task because it had a large number of items (432 in total). The correlations were high so that analyses were conducted on the total number of trials.

3 The Reading span task was administered twice, at one week intervals, to test for possible retest effects. Performance was very similar; consequently, scores were computed on 20 items by condition.

4 In these analyses, we analyzed the number of correctly recalled words/position by trial and not the percentage of success on the task (often used as an accuracy score). This allows the computing of a standard deviation across trials (20 trials by condition for reading span and 10 trials by condition for matrices task), but is of course computed on a range that is smaller in low span individuals. Note also that the errors in recall were, for the most part, omissions.

5 Analyses on the coefficient of variation (CV) have also been conducted, the results on the reaction times (RTs) score are substantially the same as those obtained with the standard deviations. However, the use of CV has often been criticized, on the basis of our ignorance of its distribution. More importantly, we did not use CV because we wanted to use the same indices on both types of measures, namely, RTs and accuracy. Indeed, Golay, Fagot & Lecerf (2013) showed that the CV cannot be used, or at least generates problems, on accuracy data. Contrary to RTs, accuracy data have a lower and an upper bound, whereas RTs have only a lower bound. Moreover, these authors also showed that the CV is influenced by the number of items which is an issue when dealing with missing data. Therefore the intra-individual standard deviations seemed more appropriate than CVs.

6 Remember that the WM tasks were not strictly identical for all participants, as they were adapted to their individual span (see Table A2). This is an optimal solution given the range of age and individual differences in the sample: If the task had been strictly identical for all, it would have been much too difficult for children (or too easy for younger adults), and would have tapped a very different capability while also yielding discouragement in the participants. This was not a problem for the RT tasks, as most of them are very simple. Consequently, age differences in the mean level of WM tasks might reflect both the level of the task and the participant’s WM capacity. Using a percentage score (e.g., ratio of the correctly remembered words on the mean difficulty presented) would not provide much relevant information because it would simply reflect how well the task is adapted to the individuals’ capacity.

7 Additional analyses were conducted on RTs, by refining the age group comparisons (see Table A1). First, these analyses showed that whichever the task, young children (9–10 years) were slower and more variable than older children (11–12 years). Second, for SRT, LI, CC and DI tasks, no difference between young-old adults (<70 years) and old-old adults (≥70 years) was obtained on iM. For LC and ST tasks, young-old adults were faster than old-old adults. As concerns the iSDr analyses, except for the LC task, young-old adults were less variable than old-old adults (see Table A3 for more details).

8 Additional analyses, refining the age group comparisons, were conducted on accuracy scores. First, the analyses of iM showed that whichever the task, young children (9–10 years) recalled fewer items than older children (11–12 years). No significant difference between these two groups was obtained for iSDr in all tasks. Second, young-old adults recalled more items than old-old adults only for Matrices task word-position associations. No significant difference between young-old and old-old adults was obtained for iSDr in any task (see Table A4 for more details).

## Appendix A

**Table A1:** Participants’ Characteristics by Age Group.

	Age Groups									
	Children				Adults					
	Young		Old		Young		Young-Old		Old-Old	
N	100		101		137		117		102	
Female	38		54		117		90		75	
	**M**	**SD**	**M**	**SD**	**M**	**SD**	**M**	**SD**	**M**	**SD**
Age	9.5	0.50	11.50	0.50	21.71	2.53	64.82	2.68	76.15	4.65
Vocabulary Score	-	-	-	-	34.67	3.25	38.04	4.38	37.37	4.91
Raven	34.14	8.39	39.51	7.11	52.15	4.91	39.53	7.54	32.90	9.54

Note: M: Group’s mean; SD: Group’s standard deviations.

**Table A2:** Participants’ span level (preliminary phase) by Task and by Age Group.

		Children	Young Adults	Older Adults
Rspan task Span level	Min	2	2	2
	Max	5	6	6
	M	2.39	3.15	2.85
	SD	0.63	0.90	0.90
Matrices task Span level	Min	2	2	2
	Max	7	7	7
	M	3.4	5.71	3.91
	SD	1.45	1.45	1.23

Note: Min: Minimum; Max: Maximum; M: Group’s mean; SD: Group’s standard deviations.

**Table A3:** Reaction time tasks: Means (iM) and intra-individual standard deviation of residual scores (iSDr) by Age group (five groups).

			Age Groups									
			Children				Adults					
			Young		Old		Young		Young-Old		Old-Old	
			M	SD	M	SD	M	SD	M	SD	M	SD
iM	SRT		381.92	74.88	338.35	54.38	272.89	39.16	324.42	76.48	343.91	72.95
	LI		605.34	97.71	525.81	88.85	372.38	43.53	446.26	64.32	470.75	75.10
	CC		536.80	106.33	454.75	87.46	325.69	39.26	417.52	68.44	446.66	82.86
	DI		1924.67	396.48	1551.83	419.32	1040.56	221.47	1569.50	302.43	1783.05	372.95
	LC	6 letters	4038.80	1323.61	3213.18	385.40	1838.17	423.31	2771.18	624.07	3200.73	912.54
		9 letters	5493.01	1646.70	4687.25	1336.96	2798.36	654.47	4029.01	950.60	4646.51	1198.88
	ST	Neutral	885.56	147.50	799.29	133.41	594.78	75.85	694.66	93.16	758.45	118.29
		Incongruent	1074.46	188.02	965.99	165.44	720.89	102.31	875.75	137.56	987.62	177.51
iSDr	SRT		12.18	2.94	10.60	2.94	7.16	2.15	8.64	2.31	9.90	3.06
	LI		13.23	3.59	11.27	3.10	6.42	1.98	7.97	2.11	9.29	3.15
	CC		13.58	3.91	10398	3.54	5.93	1.78	8.25	2.27	9.56	2.57
	DI		13.42	3.08	10.68	3.47	5.69	1.91	8.74	2.39	9.82	2.18
	LC	6 letters	14.38	5.29	10.71	4.47	5.03	1.67	7.70	2.64	8.72	3.01
		9 letters	14.01	3.93	11.25	3.84	5.83	1.81	8.38	2.30	9.44	2.72
	ST	Neutral	14.03	3.61	11.91	3.80	6.16	1.91	7.03	2.26	8.49	3.17
		Incongruent	13.22	2.95	11.59	2.73	7.16	1.92	8.24	2.33	9.52	2.62

Note: SRT: Simple reaction time task, LI: Line comparison task; CS: Cross-square task; LC: Letter comparison task; DI: Digit symbol task; ST: Stroop task; iM: intra-individual mean; iSDr: standard deviation of residual scores; M: Group’s mean; SD: Group’s standard deviations.

**Table A4:** Accuracy tasks: Means (iM) and intra-individual standard deviation of residual scores (iSDr) by Age group (five groups).

			Age Groups									
			Children				Adults					
			Young		Old		Young		Young-Old		Old-Old	
			M	SD	M	SD	M	SD	M	SD	M	SD
iM	Reading span	*n*	1.92	0.41	2.22	0.59	2.83	0.76	2.59	0.82	2.31	0.67
		*n* + 1	2.12	0.47	2.52	0.61	3.27	0.69	2.95	0.81	2.68	0.69
	Matrices tasks—Positions	*n*	3.04	1.19	3.66	1.40	5.32	1.41	3.41	1.17	3.16	0.97
		*n* + 1	3.66	1.28	4.40	1.57	6.14	1.53	3.98	1.17	3.64	0.97
	Matrices tasks—Associations	*n* + 1	1.98	0.70	2.52	0.54	3.24	0.63	2.65	0.66	2.33	0.59
		*n* + 2	1.98	0.65	2.53	0.64	3.30	0.75	2.50	0.77	2.21	0.63
iSDr	Reading span	*n*	8.09	2.66	8.70	3.75	9.20	4.84	9.71	4.71	9.98	4.40
		*n* + 1	9.26	2.00	9.85	2.38	10.14	2.90	10.18	2.81	9.96	2.69
	Matrices tasks—Positions	*n*	9.32	4.53	10.0	4.97	8.49	6.03	9.28	4.98	8.94	4.99
		*n* + 1	9.67	3.62	9.08	3.80	8.92	5.02	10.34	4.57	10.1	4.0
	Matrices tasks—Associations	*n* + 1	9.01	2.58	9.97	2.64	11.28	3.40	9.73	3.59	9.82	3.03
		*n* + 2	8.93	2.49	9.77	2.47	11.49	3.42	10.08	2.80	9.87	2.49

Note: iM: intra-individual mean; iSDr: standard deviation of residual scores; M: Group’s mean; SD: Group’s standard deviations.
